# Avoidance of Blood Transfusion to Patients Suffering From Myocardial Injury and Severe Anemia Is Associated With Increased Long-Term Mortality: Erratum

**DOI:** 10.1097/01.md.0000504795.30089.80

**Published:** 2016-10-21

In the article “Avoidance of Blood Transfusion to Patients Suffering From Myocardial Injury and Severe Anemia Is Associated With Increased Long-Term Mortality”,^[1]^ which appeared in Volume 94, Issue 38 of *Medicine*, one of Dr. Robert's affiliations was not originally included. The article has since been corrected online.
